# Identification of Immune-Related Genes Concurrently Involved in Critical Illnesses Across Different Etiologies: A Data-Driven Analysis

**DOI:** 10.3389/fimmu.2022.858864

**Published:** 2022-05-09

**Authors:** Yaojun Peng, Qiyan Wu, Qing Zhou, Zhanglin Yang, Fan Yin, Lingxiong Wang, Qi Chen, Cong Feng, Xuewen Ren, Tianyi Liu

**Affiliations:** ^1^ Department of Emergency, The First Medical Center, Chinese People's Liberation Army (PLA) General Hospital, Beijing, China; ^2^ Institute of Oncology, The Fifth Medical Centre, Chinese People's Liberation Army (PLA) General Hospital, Beijing, China; ^3^ Department of Gastroenterology, The Second Medical Center, Chinese People's Liberation Army (PLA) General Hospital, Beijing, China; ^4^ Department of Oncology, The Second Medical Center & National Clinical Research Center of Geriatric Disease, Chinese People's Liberation Army (PLA) General Hospital, Beijing, China; ^5^ Department of Traditional Chinese Medicine, The First Medical Center, Chinese People's Liberation Army (PLA) General Hospital, Beijing, China

**Keywords:** critical illness, trauma, sepsis, WGCNA, data-driven analysis, immune response

## Abstract

Severe trauma and sepsis can lead to multiple organ dysfunction syndrome, which is a leading cause of death in intensive care units with mortality rates in excess of 50%. In addition to infection, the degree of immuno-inflammatory response also influences the outcome. The genomic changes observed after a variety of pathophysiological insults, such as trauma, sepsis, burns are similar, and consist of innate immune activation and adaptive immunity suppression. However, the characteristics of the shared mechanisms of aforementioned critical illnesses and the clinical relevance remain less explored. In the present study, we performed a data analysis to identify functional genes concurrently involved in critical illnesses across differing etiologies (trauma and sepsis derived from community-acquired pneumonia/abdominal source) and explored the shared signaling pathways these common genes involved in to gain insight into the underlying molecular mechanisms. A number of immune-related biological functions were found to be dysregulated in both trauma and sepsis in the present study, so we continued to identify immune-related common genes, profiled the immune cell proportion, and explored the relationships between them. The diagnostic and prognostic value of the immune-related common genes was also evaluated to address their potential clinical utilization as novel biomarkers. Notably, we identified a list of 14 immune-related genes concurrently dysregulated in trauma and sepsis showing favorable diagnostic value, among which S100P can predict prognosis of sepsis patients. Moreover, a spectrum of immune cell subsets including naïve B cells, CD8+ T cells, CD4+ memory resting T cells, activated NK cells, resting dendritic cells, plasma cells, Tregs, macrophages M0 and macrophages M1 was found to be concurrently dysregulated in both trauma and sepsis, and a close relation between above identified immune-related genes and immune cell subsets was observed. Our data-driven findings lay a foundation for future research to elucidate the pathophysiology regarding the aspect of inflammatory and immune response in critical illnesses, and suggest future studies focus on interpreting the function roles of the identified immune-related genes, as well as the reactive immune cell subsets.

## Introduction

Trauma initiates a complex immune response in the minutes after the initial insult, contributing to complications, such as the development of sepsis and multiple organ dysfunction syndrome (1). Triggered by the aberrant host response to infection, sepsis is a complex and explicitly lethal clinical syndrome that leads to respiratory failure and ultimately death (2). Despite the use of modern antibiotics and resuscitation therapies, trauma and sepsis remain the most lethal diseases in the emergency department. Trauma is one of the leading causes of mortality and disability worldwide (3), and sepsis is responsible for one out of three in-hospital deaths (4). Trauma and sepsis are common health problems that pose great challenges to critical care, with genetic variability, disease heterogeneity, and poor understanding of the underlying pathophysiology considered the main reasons for the poor prognosis. Therefore, there is an urgent need to identify biomarkers that specifically reflect the pathophysiological changes during disease course.

Studies report that trauma and sepsis share a similar pathobiological process: injured tissue and pathogens provoke a systemic inflammatory reaction that can lead to overwhelming inflammation, whereas adaptive immune suppression concurrent with the innate hyperinflammatory response can become chronic (5). Persistent immunosuppression is associated with high mortality; however, the pathophysiology leading to the immunosuppressive state remains unclear. Analysis of whole blood transcriptomics may help uncover the biological processes and underlying mechanisms of disease by identifying patient responses to a variety of pathophysiological insults. For example, whole blood transcriptomics analysis has been applied to predict outcomes in trauma (6) and clarify clinically relevant subgroups in sepsis (7). Moreover, shared transcriptomic responses have been revealed across different etiologies of sepsis and diseases, including trauma, burns, and endotoxemia. However, the characteristics of the shared mechanisms of the aforementioned critical illnesses and their clinical relevance remain less explored.

In this study, we performed a data analysis to identify functional genes concurrently involved in critical illnesses across different etiologies (trauma and sepsis derived from community-acquired pneumonia/abdominal source) and explored the shared signaling pathways of these common genes in order to tentatively unveil the underlying molecular mechanisms. We found that multiple immune-related biological functions are dysregulated in both trauma and sepsis, resulting in our subsequent identification of immune-related common genes, profiling of the immune cell proportion, and exploration of their shared relationships. Additionally, we assessed the diagnostic and prognostic value of the immune-related common genes in order to determine their potential clinical use as novel biomarkers.

## Materials and Methods

### Data Collection and Pre-Processing

Data were downloaded from the Gene Expression Omnibus (https://www.ncbi.nlm.nih.gov/geo/). GSE11375 included 158 adult patients with severe blunt trauma and 26 healthy volunteers (8). Blood samples from trauma patients were collected within 12 h of the injury and subjected to the GPL570 Affymetrix Human Genome U133 Plus 2.0 Array (HGU133_Plus_2) platform for gene-expression quantification. GSE65682 included array-based gene expression profiles of whole-blood leukocytes from 159 sepsis patients (sepsis due to community-acquired pneumonia in 108 patients or abdominal source in 51 patients) and 42 healthy volunteers (9, 10). Blood samples were taken within 24 h of admission to critical care and subjected to the GPL13667 Affymetrix Human Genome U219 Array (HG-U219) platform for gene-expression quantification. For data pre-processing, probes were first transformed into gene symbols based on the annotation files provided by the platform manufacturers. In particular, probes without corresponding gene symbols were removed, and average values were obtained if one gene corresponded to multiple probes. Two expression datasets (trauma and sepsis) were generated from the corresponding gene matrices in the form of raw gene counts, and the voom function in the limma R package (https://bioconductor.org/packages/release/bioc/html/limma.html) was used to normalize the datasets (11). The two gene-expression datasets of the trauma and sepsis cohorts were then merged and normalized to remove the batch effect using the sva R package (https://bioconductor.org/packages/release/bioc/html/sva.html) (12).

### Differential Analysis

To identify potentially functional genes involved in trauma or sepsis, differential analysis between trauma patients and healthy controls in GSE11375 or between sepsis patients and healthy controls in GSE65682 was performed using the limma R package. The screening threshold of differentially expressed genes (DEGs) was set at a log_2_ |fold change (FC)| > 1 and an adjusted (adj.) P < 0.05.

### Coexpression Analysis

Weighted gene co-expression network analysis (WGCNA) is a systematic bioinformatics method with a core algorithm that generates a weighted network and co-expression network of genes (13). WGCNA constructs a free-scale network to explore the interrelationship between genes within the corresponding gene module and associations between gene modules and clinical traits (13). Genes with expression variances in the upper 50% were selected for WGCNA analysis. First, we performed sample hierarchical clustering with the standard R function “hclust”, followed by construction of a weighted correlation network using the WGCNA R package (https://cran.r-project.org/web/packages/WGCNA/index.html) (14). For the scale-free network, the soft power of R^2^ was set depending on the scale independence and mean connectivity. Subsequently, genes with similar patterns were clustered into the same modules (minimum size = 50) using average linkage hierarchical clustering. We merged modules with highly correlated eigengenes, with a minimum module merging height of 0.25. Gene significance and module membership were calculated to validate the stability, and correlations between clinical traits and gene modules were explored using Pearson’s correlation analysis and displayed in a heatmap plot. Modules with the highest positive correlation coefficient were considered as potential modules that were highly associated with clinical traits, with these modules chosen for further analysis.

### Intersection Analysis for Common Genes and Immune-Related Common Genes in Trauma and Sepsis

We extracted genes in the most positively correlated WGCNA modules with trauma and sepsis, with these two lists of genes subjected to intersection analysis with the two lists of DEGs in trauma and sepsis to obtain the common genes shared by these two critical illnesses (downregulated and upregulated clusters). Intersection analysis was performed by drawing a Venn diagram. A list of immune-related genes was downloaded from the Immunology Database and Analysis Portal database (ImmPort; https://www.immport.org) (**Table S1**), and immune-related genes were intersected with the identified common genes by drawing a Venn diagram.

### Enrichment Analysis

We collected downregulated and upregulated common gene clusters of trauma and sepsis as input for enrichment analysis. The clusterProfiler R package (https://bioconductor.org/packages/release/bioc/html/clusterProfiler.html) was used to perform functional enrichment analysis (15). A P < 0.05 was considered statistically significant for the enrichment analysis.

### Profile of the Leukocyte Cell Fraction

CIBERSORT is a well-established technique for estimating the immune cell composition of “bulk tissue” from gene-expression data (16). In the CIBERSORT method, an input matrix of reference gene-expression signatures is established, based on which a novel application of linear support vector regression is used to estimate the relative proportions of each cell type of interest from the target microarray or RNA-seq data matrix. We then determined an empirically defined global P-value for deconvolution. Such deconvolution techniques have been applied in a range of studies to estimate immune cell composition from fresh, frozen, and fixed specimens of peripheral blood, solid tumor tissue, or unknown content. In the present study, we evaluated leukocyte cell fractions of whole blood transcriptomics using the CIBERSORT method, which was executed using R software.

### Statistical Analysis

Two-group comparisons were determined using Student’s *t* test, and multiple group comparisons were conducted using the analysis of variance *t* test. The relationship between immune-related common genes and the immune cell fraction was determined using Pearson’s correlation coefficient. To verify the diagnostic values of the immune-related common genes, we extracted the expression data of healthy controls and trauma (or sepsis) patients and performed receiver operating characteristic curve analysis. Survival differences between the two groups were assessed using the Kaplan–Meier estimate and compared using the log-rank test. All results with a two-sided P < 0.05 were considered significant. Statistical analyses were performed using R software (v.3.6.0; https://www.r-project.org/) or GraphPad Prism (v.8.0.2; GraphPad Software, La Jolla, CA, USA).

## 3 Results

### DEGs in Trauma and Sepsis

First, we merged the GSE11375 (trauma cohort) and GSE65682 (sepsis cohort) expression matrices, after which inter-batch differences were removed using the sva package. The sample distributions before and after eliminating the batch effect are shown in **Supplementary Figure 1A**. The results indicated the elimination of the batch difference. To identify DEGs between healthy controls and patients with trauma or sepsis, we employed the limma R package with a |Log_2_ (FC) | >1 and adj. P < 0.05 used as the screening criteria for DEGs. The results identified 657 significantly downregulated genes and 657 significantly upregulated genes in trauma (**Supplementary Figure 1B** and **Table S2**), whereas 485 genes were significantly downregulated, and 390 genes significantly upregulated in sepsis (**Supplementary Figure 1C** and **Table S3**).

### Key Modules Associated With Trauma and Sepsis by WGCNA

To detect the functional modules in trauma and sepsis, we established two gene co-expression networks based on the trauma and sepsis cohorts. First, samples were clustered using the “hclust” function in the WGCNA R package to detect outliers. Sample dendrograms and trait heatmaps are shown in **Supplementary Figure 2A**. The expression profile of GSM1602973 in the sepsis cohort was excluded from subsequent WGCNA analyses (cutHeight = 80). To establish the scale-free networks, soft-powers β = 16 (R^2^ = 0.954) and β = 9 (R^2^ = 0.974) were applied to the trauma and sepsis cohorts, respectively (**Supplementary Figures 2B, C**), and modules with distances of <0.25 were merged (**Supplementary Figure 2D**). The cluster dendrograms of co-expression network modules for the trauma and sepsis cohorts are shown in **Supplementary Figure 2E**. We detected six modules in the trauma cohort and 12 in the sepsis cohort, with two heatmaps displaying the relationship between these modules and clinical traits (control *vs*. trauma [or sepsis]) shown in **Supplementary Figure 2F**. The turquoise module of the GSE11375 dataset was most positively associated with trauma (Pearson r = 0.87, P = 3×10^−58^), and the brown module of the GSE65682 dataset was positively associated with sepsis (Pearson r = 0.81, P=4×10^−47^). To further evaluate the correlation between trauma (or sepsis) and the modules, we calculated module membership and gene significance. The turquoise module (Pearson r = 0.77; P = 1.2×10^−99^) showed a high positive correlation with trauma, whereas the brown module (Pearson r = 0.86; P = 7×10^−141^) showed a high positive correlation with sepsis (**Supplementary Figure 2G**).

### Common Genes and Biological Functions Shared by Trauma and Sepsis

Previous studies report that trauma and sepsis share a similar pathobiological process (5). Therefore, we selected genes in the turquoise module of the trauma cohort (*n* = 502; **Table S4**), genes in the brown module of the sepsis cohort (*n* = 477; **Table S5**), DEGs of trauma (*n* = 1314; **Table S2**), and DEGs of sepsis (*n* = 875; **Table S3**) as input genes and performed intersection analysis to identify common genes shared by these two diseases. In total, we identified 144 functional genes as concurrently involved in trauma and sepsis, including 124 upregulated and 20 downregulated genes (**Supplementary Figure 3A**). Enrichment analysis showed that the upregulated common genes were mainly enriched in pathways related to neutrophil activation and degranulation, neutrophil-mediated immunity, arginine catabolic and metabolic processes, and defense responses to bacteria and other organisms, whereas the downregulated common genes were mainly enriched in pathways related to interferon-γ production and secretion, chemokine-mediated signaling, T cell-mediated immunity, and amine metabolic processes (**Supplementary Figure 3B**).

### Immune-Related Common Genes, Immune Cell Profiles, and Their Association in Trauma and Sepsis

**Figure 1:**
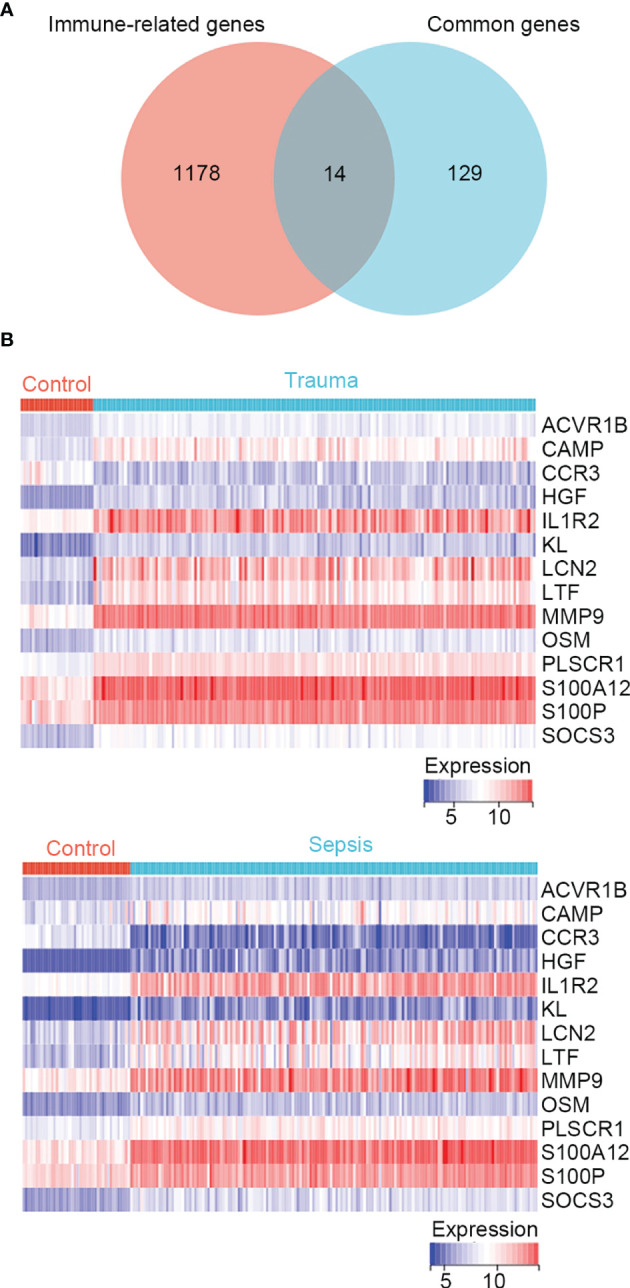
Identification of immune-related genes concurrently involved in trauma and sepsis. **(A)** The Venn diagram of genes between the immune-related genes and the identified common genes. A total of 14 overlapping immune-related common genes were detected. **(B)** Expression profiles of the 14 immune-related common genes in trauma and sepsis.

In the enrichment analysis, several immune-related biological functions were concurrently dysregulated in trauma and sepsis. Therefore, we identified immune-related common genes and assessed immune cell infiltration in patients with trauma and sepsis. A list of 1793 immune-related genes were retrieved from the ImmPort database (**Table S1**), and 14 common genes were intersected with those in the immune-related gene list (*ACVR1B, CAMP, CCR3, HGF, IL1R2, KL, LCN2, LTF, MMP9, OSM, PLSCR1, S100A12, S100P*, and *SOCS3*) (**Figure 1A** and **Table S6**). The expression of these immune-related common genes in trauma and sepsis are shown in **Figure 1B**.

**Figure 2:**
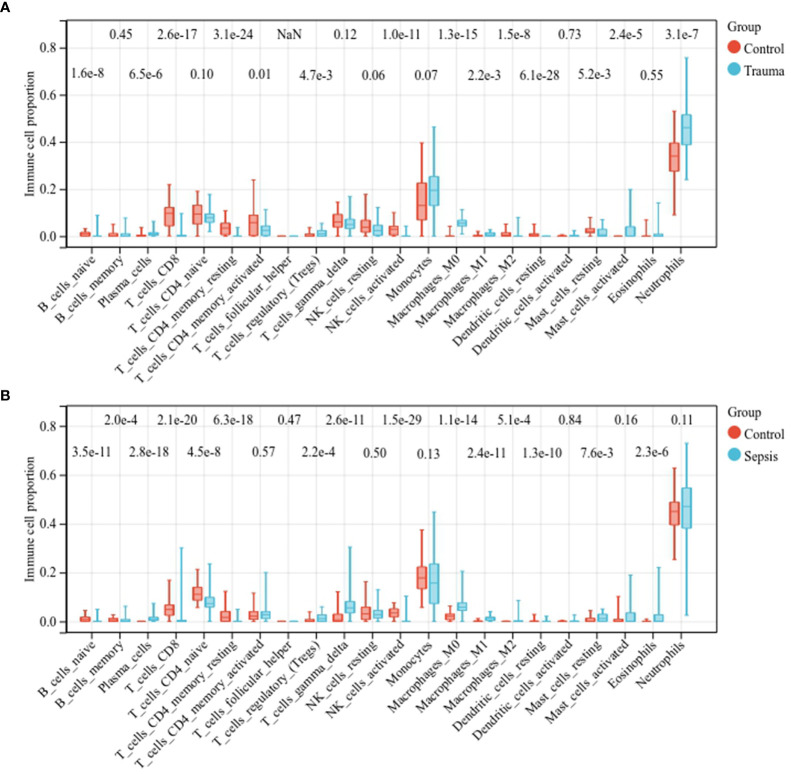
Comparison of the immune cell fractions. Comparison of immune cell fractions between **(A)** healthy controls and trauma patients and **(B)** healthy controls and sepsis patients.

We then assessed the immune cell fractions in patients with trauma and sepsis (**Supplementary Figure S4**). In trauma patients, naïve B cells, CD8+ T cells, CD4+ memory resting T cells, activated CD4+ memory T cells, activated natural killer (NK) cells, M2 macrophages, and resting dendritic cells were significantly reduced, whereas plasma cells, regulatory T cells regulatory (Tregs), M0 macrophages, M1 macrophages, resting mast cells, activated mast cells, and neutrophils were significantly upregulated (**Figure 2A**). In patients with sepsis, naïve B-cells, memory B-cells, CD8+ T cells, naïve CD4+ T cells, CD4+ memory resting T cells, activated NK cells, and resting dendritic cells were significantly downregulated, whereas plasma cells, Tregs, γδ T cells, M0 macrophages, M1 macrophages, M2 macrophages, mast cells, and eosinophils were significantly upregulated (**Figure 2B**). These results suggest a similar spectrum of immune cell fractions in trauma and sepsis patients.

**Figure 3:**
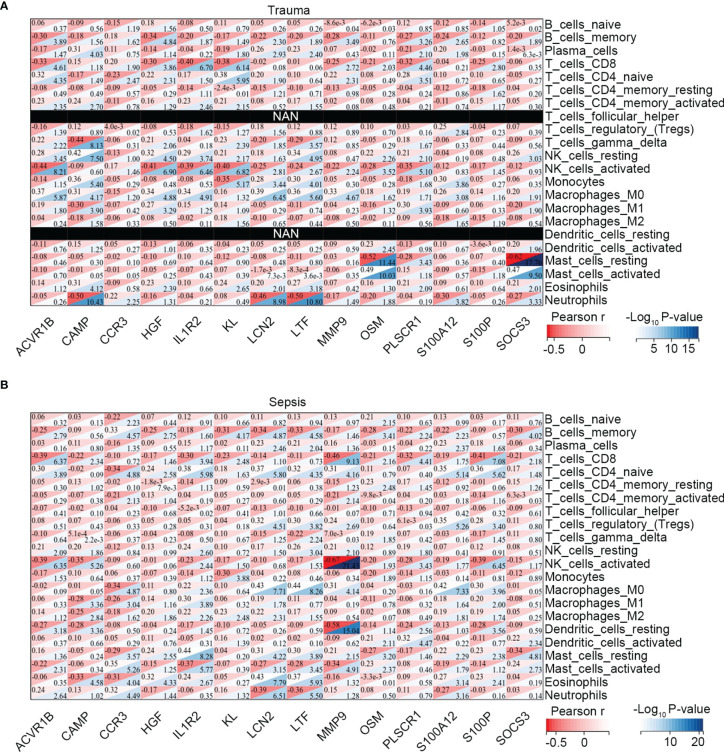
Association between the immune-related common genes and the immune cell fractions. Associations identified in **(A)** trauma, and **(B)** sepsis.

Moreover, we explored the association between immune-related common genes and immune cell fractions in trauma and sepsis patients. The results showed that the identified immune-related common genes were more or less significantly related to immune cells concurrently dysregulated in trauma and sepsis patients (**Figures 3A, B**). For example, NK cell activation was significantly upregulated in both trauma and sepsis patients, with 11 of 14 immune-related common genes (*ACVR1B, CCR3, HGF, IL1R2, KL, LCN2, LTF, MMP9, OSM, PLSCR1*, and *S100P*) significantly associated with activated NK cells in trauma patients (**Figure 3A**) and 10 of 14 immune-related common genes (*ACVR1B, CAMP, IL1R2, KL, LTF, MMP9, OSM, PLSCR1, S100A12*, and *S100P*) significantly associated with activated NK cells in sepsis patients (**Figure 3B**).

### Clinical Relevance of the Immune-Related Common Genes in Trauma and Sepsis

**Figure 4:**
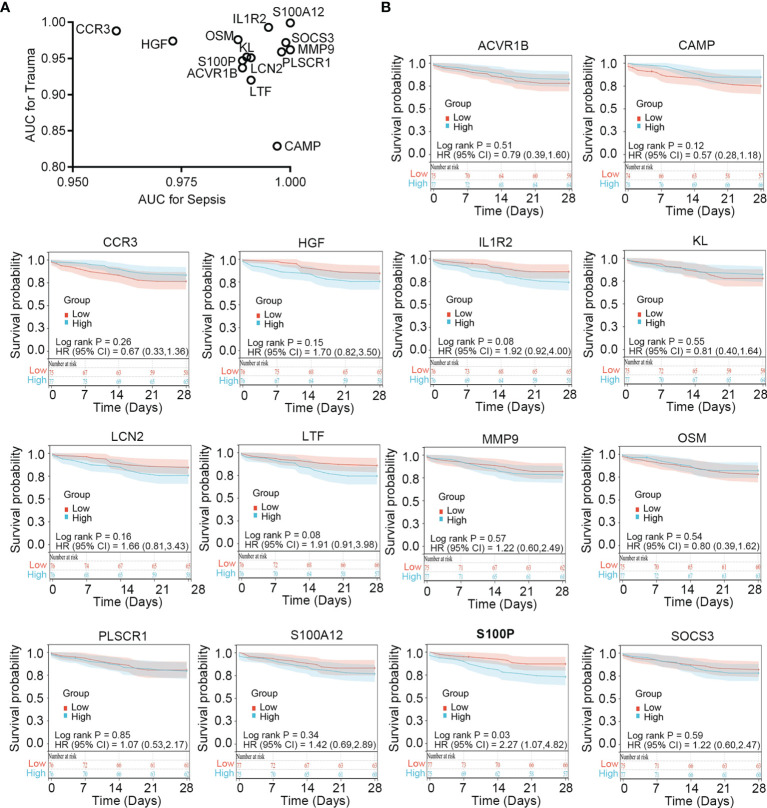
Clinical relevance of the immune-related common genes in trauma and sepsis. **(A)** ROC curve analysis of the immune-related common genes in trauma and sepsis. **(B)** Survival analysis of the immune-related common genes in sepsis. ROC, receiver operating characteristic; AUC, area under the ROC curve.

To verify the diagnostic value of the immune-related common genes, we performed receiver operating characteristic curve analysis. The results showed that these genes demonstrate favorable diagnostic potential for both trauma and sepsis (**Figure 4A**). Because 28-day follow-up information for the sepsis cohort (*n* = 152; **Table S7**) was available in the GSE65682 dataset, we performed survival analysis to explore whether these immune-related common genes show prognostic potential for sepsis. Patients showing an upper 50^th^ percentile of expression for a given gene were designated as the high-expression subgroup, whereas those showing expression for a gene in the lower 50^th^ percentile were designated as the low-expression subgroup. The results showed that *S100P* expression could stratify survival differences between the two groups (log-rank P = 0.019), suggesting *S100P* as a potential prognostic biomarker for sepsis (**Figure 4B**).

## Discussion

Severe trauma and sepsis provoke a conserved “genomic storm” that dynamically alters the leukocyte transcriptome, with the upregulation of the innate immune response and a concomitant down regulation of the adaptive immune response, and these early (within hours of tissue injury) transcriptional changes are predictive of clinical outcomes (17, 18). In this study, trauma- and sepsis-related sequencing data from blood samples were collected and subjected to bioinformatics analysis in order to explore the common molecular change shared by these two critical illnesses at the early disease stage (12 h for trauma and 24 h for sepsis). Application of WGCNA analysis to identify potential biomarkers revealed 124 and 20 genes concurrently upregulated and downregulated, respectively. The enrichment analysis showed that innate immune responses and multiple metabolic pathways were significantly upregulated, whereas adaptive immune responses were significantly downregulated. These results emphasize the critical roles of dysregulated immune responses and metabolic signaling in the pathological condition of trauma and sepsis. We identified 14 immune-related genes closely associated with trauma and sepsis (*ACVR1B, CAMP, CCR3, HGF, IL1R2, KL, LCN2, LTF, MMP9, OSM, PLSCR1, S100A12, S100P*, and *SOCS3*). Some of above genes have been previously reported as playing important roles in trauma and sepsis. For example, HGF reportedly protects mitochondrial physiology by activating mammalian target of rapamycin signaling to partially ameliorate endothelial pyroptosis and attenuate vascular endothelial injury and acute lung injury in an animal model of sepsis (19). It is also reported in a prospective observational study that elevated plasma HGF level is an indicator of poor prognosis in sepsis patients (20). Brumann et al. presented a serial, sequential investigation of human neutrophil granulocytes from major trauma patients that included evaluation of the mRNA expression profiles of *IL-10, STAT3, SOCS1*, and *SOCS3*, finding that death after trauma was associated with higher *SOCS3* mRNA levels at 6 h after trauma (21). Notably, to our knowledge, ACVR1B which is a cytokine receptor and TGF-β family member receptor, was firstly identified to be related to both trauma and sepsis in the present study. Further investigations are warranted to validate the immune-related common genes at the protein level, and functional experiments are needed to explore the underlying mechanisms.

The imbalance between the hyperimmune response and immunoparalysis contributes to the mortality of critical illnesses (22). The mechanism of immune dysfunction involves dysregulation of innate and adaptive immune responses mediated by immune cells, such as neutrophils, macrophages, dendritic cells, T cells, NK cells, and inflammatory cytokines, secreted by these cell populations (23). Therefore, we profiled immune cell fractions during trauma and sepsis. The results indicated that naïve B cells, CD8+ T cells, CD4+ memory resting T cells, activated NK cells, and resting dendritic cells were concurrently downregulated, whereas plasma cells, Tregs, M0 macrophages, and M1 macrophages were concurrently upregulated. The dichotomous role of neutrophils as the major effectors of the innate immune system is well known in inflammation and infection (24). The neutrophil/lymphocyte ratio and monocyte/high-density lipoprotein cholesterol ratio trend to an elevated level in non-surviving patients with sepsis, and evaluation of the neutrophil/lymphocyte ratio together with monocyte/high-density lipoprotein cholesterol ratio is an independent risk factor for increased mortality (25). In abdominal trauma patients, on-admission platelet/lymphocyte ratio but not neutrophil/lymphocyte ratio helps early risk stratification and timely management and predicts mortality (26). Abnormal CD4+ and CD8+ T cell responses are major components of a dysregulated acquired immune response, and the immune dysfunction of Tregs also contributes to pathogenesis (27). Improved outcomes in sepsis by increasing the heterogeneous characteristics of Tregs through intervention strategies has been reported (28). The investigation of associations between immune-related common genes and immune cell fractions in trauma and sepsis patients revealed that the identified immune-related common genes were more or less significantly related to the immune cells concurrently dysregulated in trauma and sepsis, indicating their possible roles in regulating the functions of these immune cells. We noticed that MMP9 has the greatest negative correlation with activated NK cells. IL2 mediated NK cell long-term stimulation leads to down-regulation of several MMPs (including MMP9), and importantly, to a decrease in invasion capacity (29). Elevation of MMP-9 and IDO induced by pancreatic cancer cells mediates natural killer cell dysfunction (30). Therefore, we speculated that *MMP9* might relate to activation of NK cells and play a role in NK cells anergy in sepsis and trauma. We evaluated the clinical relevance of the immune-related common genes in trauma and sepsis in order to address their potential clinical use as novel biomarkers. The results showed that all of the genes showed favorable diagnostic potential for both trauma and sepsis, and specifically, high level of *S100P* was associated with poor 28-day survival of sepsis patients. S100P is produced mainly by the central nervous system, and the elevated expression level of S100P in sepsis was found by other groups (31, 32), but its role as a prognostic biomarker for sepsis has not been previously reported. Above findings have important implications for the field of critical care and suggest future studies focus on interpreting the functional roles of the identified immune-related genes, as well as the reactive immune cell subsets.

The limitations should be acknowledged for our study. First, considering the huge potential of biomarkers for personalized medicine in critical illnesses, systems-based omics approaches which encompasses genomics, epigenetics, transcriptomics, proteomics, and metabolomics are utilized for the search of biomarkers (33). The transcriptomics analysis develops fast among the above omics technologies, and as used in our study, most transcriptomic work has focused on peripheral blood leukocytes; however, the use of other omics and the mutual verification and supplement should not be ignored. Second, since disease state changes in critical illnesses are rapid and dynamic, longitudinal studies of the molecular changes seen over time and how those changes might impact patient outcomes and response to therapies are required (34). Above are objectively technological limitations related to all omics research. The authors foresee a future for the advancement of technology capable of rapid bedside tests with limited hands-on time and no need for specialized laboratories, and the development of statistical methods to analyze gene expression data over time, as well as the utilization of dynamic mathematical models of critical illness which may help resolve these problems. Finally, we have limited experimental data and lack information on the regulatory mechanisms and functional roles of the dysregulated immune genes and immune cells. Further studies are required to confirm the association of the dysregulated immune genes and immune cells with MODS in critical illnesses in a temporal setting.

In conclusion, we used integrated network analysis to identify immune-related common genes concurrently involved in trauma and sepsis, explore their relationship with the immune cell fractions, and evaluate their diagnostic and prognostic value in trauma and sepsis patients. These data-driven findings lay a foundation for future research to elucidate the pathophysiology of inflammatory and immune responses in critical illnesses.

## Data Availability Statement

The datasets presented in this study can be found in online repositories. The names of the repository/repositories and accession number(s) can be found in the article/**Supplementary Material**.

## Author Contributions

YP and TL: conceptualization. YP, ZY, FY, and LW: data curation. YP, QW, and QZ: formal analysis. YP, CF, XR, and TL: methodology. CF, XR, and TL: supervision. All authors participated in writing of the original draft of the manuscript. CF, XR, and TL: writing (review and editing). All authors contributed to the article and approved the submitted version.

## Funding

This work was supported by the National Natural Science Foundation of China (No. 82072200). The funding organizations had no role in the design and conduct of the study; the collection, management, analysis, and interpretation of the data; or the preparation and approval of the manuscript.

## Conflict of Interest

The authors declare that the research was conducted in the absence of any commercial or financial relationships that could be construed as a potential conflict of interest.

## Publisher’s Note

All claims expressed in this article are solely those of the authors and do not necessarily represent those of their affiliated organizations, or those of the publisher, the editors and the reviewers. Any product that may be evaluated in this article, or claim that may be made by its manufacturer, is not guaranteed or endorsed by the publisher.

## Supplementary Material

Supplementary Figure 1 Differential expression analysis of genes associated with trauma and sepsis. **(A)** The distribution of gene expression before and after eliminating the batch effect. **(B)** Volcano plot of DEGs in trauma and the number of downregulated and upregulated DEGs. **(C)** Volcano plot of DEGs in sepsis and the number of downregulated and upregulated DEGs. FC, fold change; FDR, false discovery rate; DEGs, differentially expressed genes.

Supplementary Figure 2 Identification of modules related to clinical traits in trauma and sepsis by WGCNA. **(A)** Sample clustering to detect outliers, excluding GSM1602973 of the sepsis cohort. **(B)** Analysis of the scale-free fit index for various soft-threshold powers. **(C)** Analysis of the mean connectivity for various soft-threshold powers. **(D)** Clustering of module eigengenes. Modules whose distance were less than 0.25 (red line) were merged. **(E)** Cluster dendrogram of co-expression network modules ordered by a hierarchical clustering of genes and based on the 1-TOM matrix. Different colors represent different modules. **(F)** Module–trait relationships. Each row represents a color module, and each column represents a clinical trait (normal and tumor). Each cell contains the corresponding correlation and P-value. **(G)** MM versus GS plots of the key modules. ME, module eigengenes; MM, module membership; GS, gene significance.

Supplementary Figure 3 Identification of common genes and biological functions shared by trauma and sepsis. **(A)** The Venn diagram of genes among the two DEG lists and the two lists of co-expressed genes. A total of 144 overlapping differentially co-expressed genes were detected (124 genes upregulated and 20 genes downregulated). **(B)** Enrichment analysis of the identified common genes.

Supplementary Figure 4 Immune cell fractions in trauma and sepsis patients.

## Abbreviations

Tregs, regulatory T cells; DEGs, differentially expressed genes; FC, fold change; WGCNA, weighted gene co-expression network analysis.
