# Supplementary figures and images for: Identification of Immune-Related Genes Concurrently Involved in Critical Illnesses Across Different Etiologies: A Data-Driven Analysis
Source: Front Immunol. 2022 May 9;13:858864. doi: 10.3389/fimmu.2022.858864 (PMC9124755; doi:10.3389/fimmu.2022.858864)

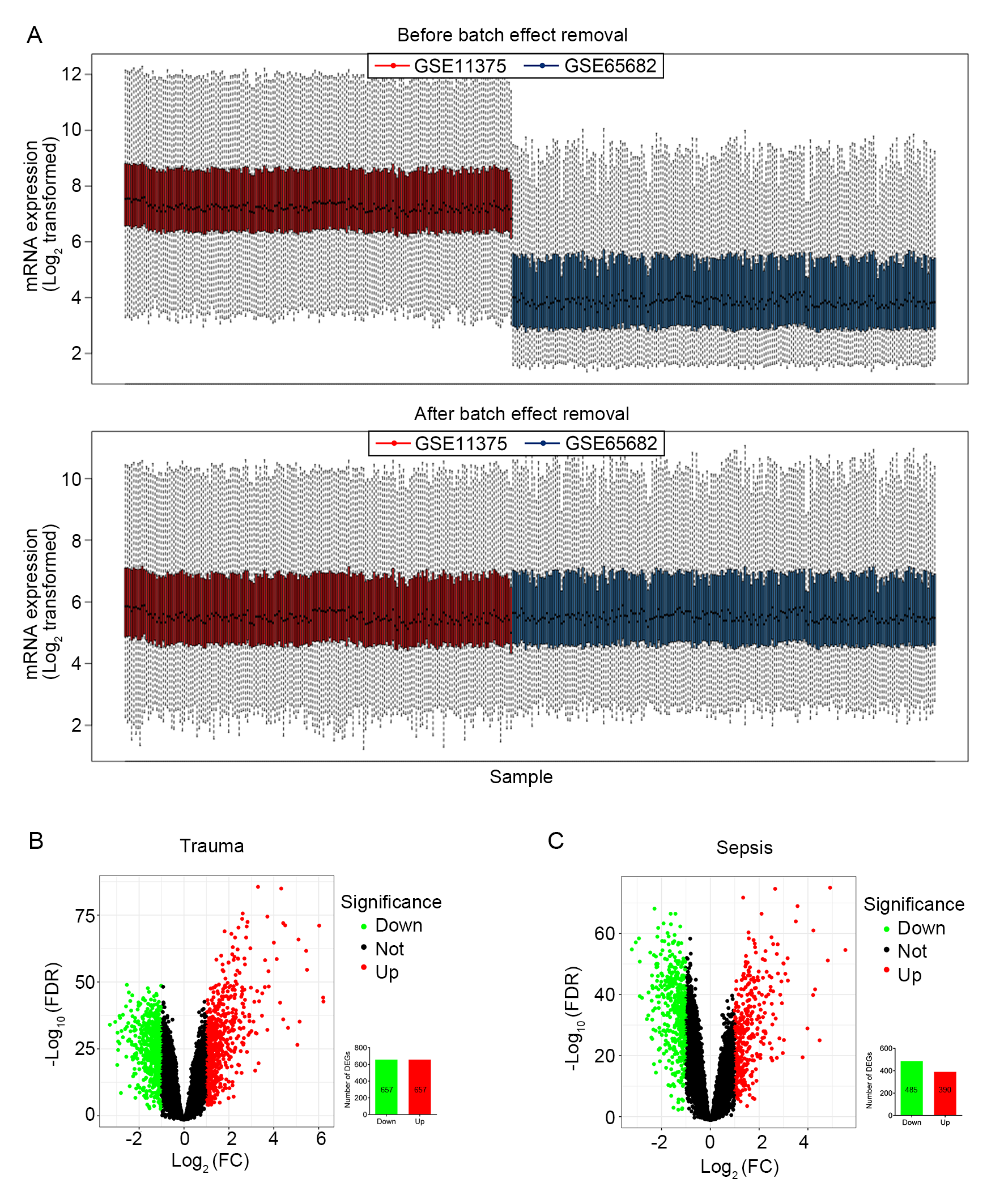

Supplement: Supplementary Figure 1 — Differential expression analysis of genes associated with trauma and sepsis. (A) The distribution of gene expression before and after eliminating the batch effect. (B) Volcano plot of DEGs in trauma and the number of downregulated and upregulated DEGs. (C) Volcano plot of DEGs in sepsis and the number of downregulated and upregulated DEGs. FC, fold change; FDR, false discovery rate; DEGs, differentially expressed genes. [file Image_1.tif]

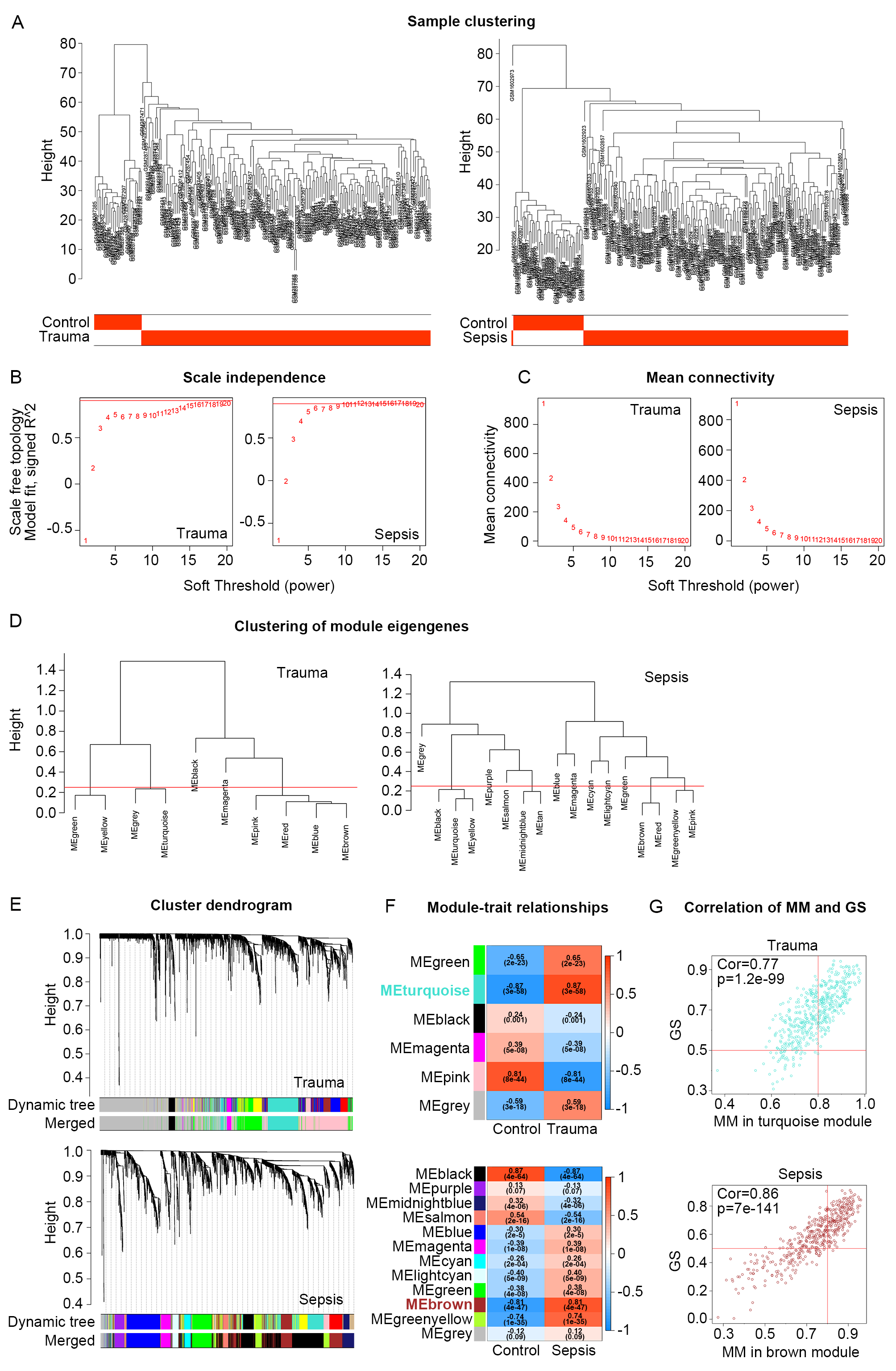

Supplement: Supplementary Figure 2 — Identification of modules related to clinical traits in trauma and sepsis by WGCNA. (A) Sample clustering to detect outliers, excluding GSM1602973 of the sepsis cohort. (B) Analysis of the scale-free fit index for various soft-threshold powers. (C) Analysis of the mean connectivity for various soft-threshold powers. (D) Clustering of module eigengenes. Modules whose distance were less than 0.25 (red line) were merged. (E) Cluster dendrogram of co-expression network modules ordered by a hierarchical clustering of genes and based on the 1-TOM matrix. Different colors represent different modules. (F) Module–trait relationships. Each row represents a color module, and each column represents a clinical trait (normal and tumor). Each cell contains the corresponding correlation and P-value. (G) MM versus GS plots of the key modules. ME, module eigengenes; MM, module membership; GS, gene significance. [file Image_2.tif]

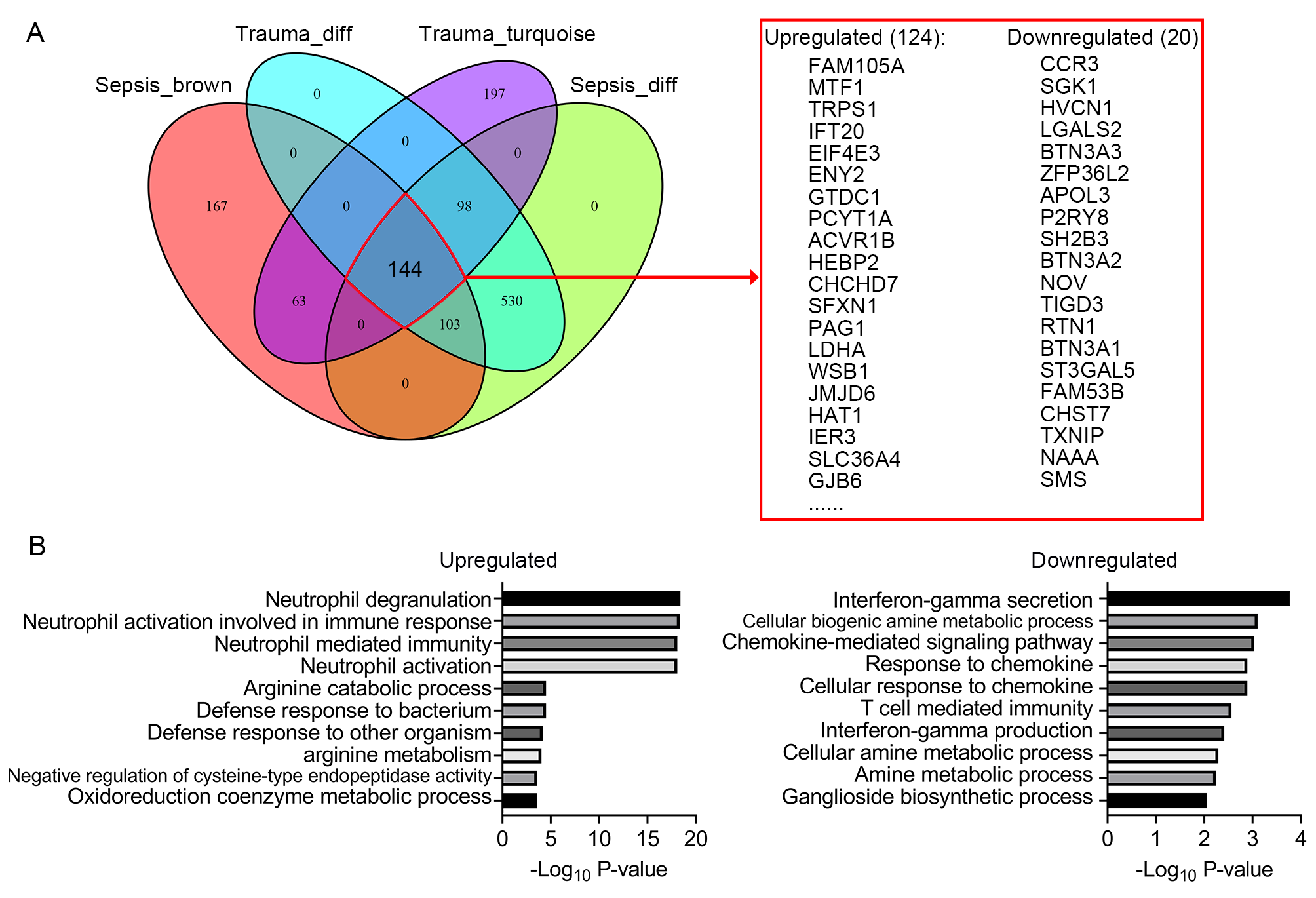

Supplement: Supplementary Figure 3 — Identification of common genes and biological functions shared by trauma and sepsis. (A) The Venn diagram of genes among the two DEG lists and the two lists of co-expressed genes. A total of 144 overlapping differentially co-expressed genes were detected (124 genes upregulated and 20 genes downregulated). (B) Enrichment analysis of the identified common genes. [file Image_3.tif]

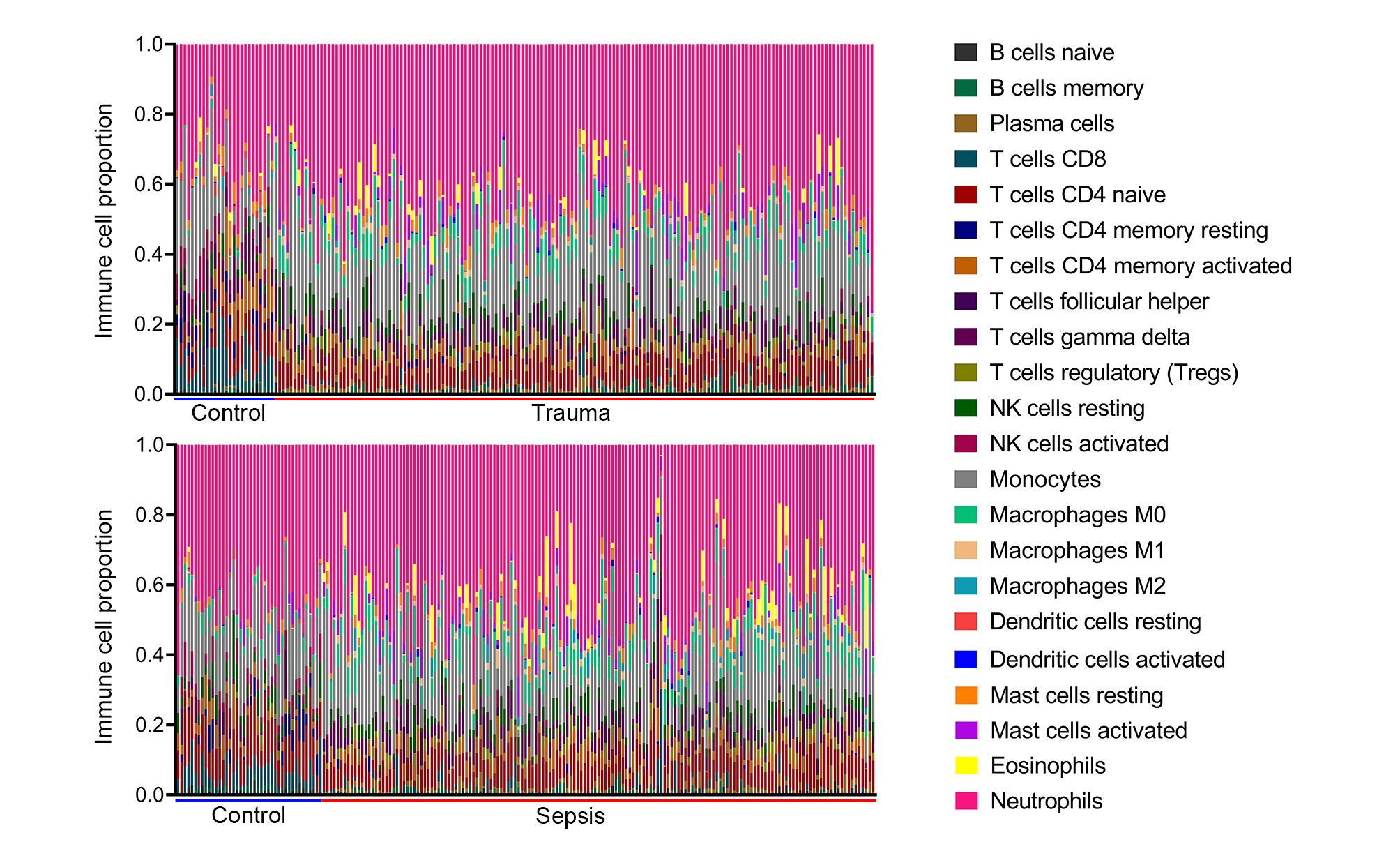

Supplement: Supplementary Figure 4 — Immune cell fractions in trauma and sepsis patients. [file Image_4.tif]
