# Supplementary material for: Identification of Immune-Related Genes Concurrently Involved in Critical Illnesses Across Different Etiologies: A Data-Driven Analysis
Source: Front Immunol. 2022 May 9;13:858864. doi: 10.3389/fimmu.2022.858864 (PMC9124755; doi:10.3389/fimmu.2022.858864)
Supplement: Supplementary file 10 [file Table_6.docx]

**Table S6.** The identified 14 immune-related genes concurrently dysregulated in trauma and sepsis.

| Gene symbol | Description | Chr location | Category | Associated with trauma and sepsis  (representative references) |
| --- | --- | --- | --- | --- |
| ACVR1B | activin A receptor type 1B | 12q13.13 | Cytokine receptors,  TGF-β family member receptors | Trauma: NR  Sepsis: NR |
| CAMP | cathelicidin antimicrobial peptide | 3p21.31 | Antimicrobials, Chemokines, Cytokines | Trauma: NR  Sepsis: (1) |
| CCR3 | C-C motif chemokine receptor 3 | 3p21.31 | Antimicrobials, Chemokine receptors, Cytokine receptors | Trauma: (2)  Sepsis: (3) |
| HGF | hepatocyte growth factor | 7q21.11 | Antimicrobials, Cytokines | Trauma: (4, 5)  Sepsis: (6-8) |
| IL1R2 | interleukin 1 receptor type 2 | 2q11.2 | Cytokine receptors,  Interleukins receptors | Trauma: (9)  Sepsis: (10-12) |
| KL | klotho | 13q13.1 | Cytokines | Trauma: (13)  Sepsis: (14, 15) |
| LCN2 | lipocalin 2 | 9q34.11 | Antimicrobials | Trauma: (16, 17)  Sepsis: (18-20) |
| LTF | lactotransferrin | 3p21.31 | Antimicrobials | Trauma: (21)  Sepsis: (20, 22) |
| MMP9 | matrix metallopeptidase 9 | 20q13.12 | Antimicrobials | Trauma: (23, 24)  Sepsis: (23, 25) |
| OSM | oncostatin M | 22q12.2 | Cytokines | Trauma: (26)  Sepsis: (27, 28) |
| PLSCR1 | phospholipid scramblase 1 | 3q24 | Antimicrobials | Trauma: NR  Sepsis: (26) |
| S100A12 | S100 calcium binding protein A12 | 1q21.3 | Antimicrobials | Trauma: (29, 30)  Sepsis: (31, 32) |
| S100P | S100 calcium binding protein P | 4p16.1 | Antimicrobials | Trauma: NR  Sepsis: (33) |
| SOCS3 | suppressor of cytokine signaling 3 | 17q25.3 | Antimicrobials | Trauma: (34, 35)  Sepsis: (36, 37) |

Chr, chromosome; NR, not reported.

**References**

1. Barbeiro DF, Barbeiro HV, Zampieri FG, César Machado MC, Torggler Filho F, Gomes Cunha DM, et al. Cathelicidin LL-37 bloodstream surveillance is down regulated during septic shock. Microbes and infection. 2013;15(5):342-6.

2. Ferreira AM, Rollins BJ, Faunce DE, Burns AL, Zhu X, Dipietro LA. The effect of MCP-1 depletion on chemokine and chemokine-related gene expression: evidence for a complex network in acute inflammation. Cytokine. 2005;30(2):64-71.

3. Venet F, Lepape A, Debard AL, Bienvenu J, Bohé J, Monneret G. The Th2 response as monitored by CRTH2 or CCR3 expression is severely decreased during septic shock. Clinical immunology (Orlando, Fla). 2004;113(3):278-84.

4. Loftus TJ, Kannan KB, Mira JC, Brakenridge SC, Efron PA, Mohr AM. Modulation of the HGF/c-Met Axis Impacts Prolonged Hematopoietic Progenitor Mobilization Following Trauma and Chronic Stress. Shock (Augusta, Ga). 2020;54(4):482-7.

5. Fukushima T, Uchiyama S, Tanaka H, Kataoka H. Hepatocyte Growth Factor Activator: A Proteinase Linking Tissue Injury with Repair. International journal of molecular sciences. 2018;19(11).

6. Jekarl DW, Kim JY, Ha JH, Lee S, Yoo J, Kim M, et al. Diagnosis and Prognosis of Sepsis Based on Use of Cytokines, Chemokines, and Growth Factors. Disease markers. 2019;2019:1089107.

7. Peng F, Liang C, Chang W, Sun Q, Xie J, Qiu H, et al. Prognostic Significance of Plasma Hepatocyte Growth Factor in Sepsis. Journal of intensive care medicine. 2022;37(3):352-8.

8. Peng F, Chang W, Sun Q, Xu X, Xie J, Qiu H, et al. HGF alleviates septic endothelial injury by inhibiting pyroptosis via the mTOR signalling pathway. Respiratory research. 2020;21(1):215.

9. Thompson CM, Park CH, Maier RV, O'Keefe GE. Traumatic injury, early gene expression, and gram-negative bacteremia. Critical care medicine. 2014;42(6):1397-405.

10. McCall CE, Grosso-Wilmoth LM, LaRue K, Guzman RN, Cousart SL. Tolerance to endotoxin-induced expression of the interleukin-1 beta gene in blood neutrophils of humans with the sepsis syndrome. The Journal of clinical investigation. 1993;91(3):853-61.

11. Martínez-Paz P, Aragón-Camino M, Gómez-Sánchez E, Lorenzo-López M, Gómez-Pesquera E, Fadrique-Fuentes A, et al. Distinguishing septic shock from non-septic shock in postsurgical patients using gene expression. The Journal of infection. 2021;83(2):147-55.

12. Ahmad S, Singh P, Sharma A, Arora S, Shriwash N, Rahmani AH, et al. Transcriptome Meta-Analysis Deciphers a Dysregulation in Immune Response-Associated Gene Signatures during Sepsis. Genes. 2019;10(12).

13. Liu XH, Graham ZA, Harlow L, Pan J, Azulai D, Bauman WA, et al. Spinal Cord Injury Reduces Serum Levels of Fibroblast Growth Factor-21 and Impairs Its Signaling Pathways in Liver and Adipose Tissue in Mice. Frontiers in endocrinology. 2021;12:668984.

14. Jorge LB, Coelho FO, Sanches TR, Malheiros D, Ezaquiel de Souza L, Dos Santos F, et al. Klotho deficiency aggravates sepsis-related multiple organ dysfunction. American journal of physiology Renal physiology. 2019;316(3):F438-f48.

15. Chen X, Tong H, Chen Y, Chen C, Ye J, Mo Q, et al. Klotho ameliorates sepsis-induced acute kidney injury but is irrelevant to autophagy. OncoTargets and therapy. 2018;11:867-81.

16. Braga A, Bandiera S, Verheyen J, Hamel R, Rutigliani C, Edenhofer F, et al. Combination of In Situ Lcn2 pRNA-RNAi Nanotherapeutics and iNSC Transplantation Ameliorates Experimental SCI in Mice. Molecular therapy : the journal of the American Society of Gene Therapy. 2020;28(12):2677-90.

17. Suk K. Lipocalin-2 as a therapeutic target for brain injury: An astrocentric perspective. Progress in neurobiology. 2016;144:158-72.

18. Casas A, Jr., Hawisher D, De Guzman CB, Bickler SW, De Maio A, Cauvi DM. Regulation of the Nfkbiz Gene and Its Protein Product IkBζ in Animal Models of Sepsis and Endotoxic Shock. Infection and immunity. 2021;89(4).

19. Macdonald SPJ, Bosio E, Neil C, Arendts G, Burrows S, Smart L, et al. Resistin and NGAL are associated with inflammatory response, endothelial activation and clinical outcomes in sepsis. Inflammation research : official journal of the European Histamine Research Society [et al]. 2017;66(7):611-9.

20. Almansa R, Ortega A, Ávila-Alonso A, Heredia-Rodríguez M, Martín S, Benavides D, et al. Quantification of Immune Dysregulation by Next-generation Polymerase Chain Reaction to Improve Sepsis Diagnosis in Surgical Patients. Annals of surgery. 2019;269(3):545-53.

21. Li Z, Wang Q, Yu H, Zou K, Xi Y, Mi W, et al. Screening of Key Genes in Severe Burn Injury at Different Stages via Analyzing Gene Expression Data. Journal of burn care & research : official publication of the American Burn Association. 2016;37(3):e254-62.

22. Moreno-Navarrete JM, Comas F, de Jager V, Fernández-Real JM, Bouma HR. Cecal Ligation and Puncture-Induced Sepsis Promotes Brown Adipose Tissue Inflammation Without Any Impact on Expression of Thermogenic-Related Genes. Frontiers in physiology. 2021;12:692618.

23. Teng L, Yu M, Li JM, Tang H, Yu J, Mo LH, et al. Matrix metalloproteinase-9 as new biomarkers of severity in multiple organ dysfunction syndrome caused by trauma and infection. Molecular and cellular biochemistry. 2012;360(1-2):271-7.

24. Pijet B, Stefaniuk M, Kostrzewska-Ksiezyk A, Tsilibary PE, Tzinia A, Kaczmarek L. Elevation of MMP-9 Levels Promotes Epileptogenesis After Traumatic Brain Injury. Molecular neurobiology. 2018;55(12):9294-306.

25. Gäddnäs FP, Sutinen MM, Koskela M, Tervahartiala T, Sorsa T, Salo TA, et al. Matrix-metalloproteinase-2, -8 and -9 in serum and skin blister fluid in patients with severe sepsis. Critical care (London, England). 2010;14(2):R49.

26. Slaets H, Nelissen S, Janssens K, Vidal PM, Lemmens E, Stinissen P, et al. Oncostatin M reduces lesion size and promotes functional recovery and neurite outgrowth after spinal cord injury. Molecular neurobiology. 2014;50(3):1142-51.

27. Gong Y, Yan X, Sun X, Chen T, Liu Y, Cao J. Oncostatin M Is a Prognostic Biomarker and Inflammatory Mediator for Sepsis. The Journal of infectious diseases. 2020;221(12):1989-98.

28. Guillet C, Fourcin M, Chevalier S, Pouplard A, Gascan H. ELISA detection of circulating levels of LIF, OSM, and CNTF in septic shock. Annals of the New York Academy of Sciences. 1995;762:407-9.

29. Feng MJ, Ning WB, Wang W, Lv ZH, Liu XB, Zhu Y, et al. Serum S100A12 as a prognostic biomarker of severe traumatic brain injury. Clinica chimica acta; international journal of clinical chemistry. 2018;480:84-91.

30. Petrone AB, Gionis V, Giersch R, Barr TL. Immune biomarkers for the diagnosis of mild traumatic brain injury. NeuroRehabilitation. 2017;40(4):501-8.

31. Zhang Z, Han N, Shen Y. S100A12 promotes inflammation and cell apoptosis in sepsis-induced ARDS via activation of NLRP3 inﬂammasome signaling. Molecular immunology. 2020;122:38-48.

32. Achouiti A, Föll D, Vogl T, van Till JW, Laterre PF, Dugernier T, et al. S100A12 and soluble receptor for advanced glycation end products levels during human severe sepsis. Shock (Augusta, Ga). 2013;40(3):188-94.

33. Lu J, Li Q, Wu Z, Zhong Z, Ji P, Li H, et al. Two gene set variation indexes as potential diagnostic tool for sepsis. American journal of translational research. 2020;12(6):2749-59.

34. Park KW, Lin CY, Lee YS. Expression of suppressor of cytokine signaling-3 (SOCS3) and its role in neuronal death after complete spinal cord injury. Experimental neurology. 2014;261:65-75.

35. Brumann M, Matz M, Kusmenkov T, Stegmaier J, Biberthaler P, Kanz KG, et al. Impact of STAT/SOCS mRNA expression levels after major injury. Mediators of inflammation. 2014;2014:749175.

36. Fang M, Dai H, Yu G, Gong F. Gene delivery of SOCS3 protects mice from lethal endotoxic shock. Cellular & molecular immunology. 2005;2(5):373-7.

37. Grutkoski PS, Chen Y, Chung CS, Ayala A. Sepsis-induced SOCS-3 expression is immunologically restricted to phagocytes. Journal of leukocyte biology. 2003;74(5):916-22.
